# Health Care Professionals’ Concerns About Medical AI and Psychological Barriers and Strategies for Successful Implementation: Scoping Review

**DOI:** 10.2196/66986

**Published:** 2025-04-23

**Authors:** Nora Arvai, Gellért Katonai, Bertalan Mesko

**Affiliations:** 1 Kálmán Laki Doctoral School of Biomedical and Clinical Sciences University of Debrecen Debrecen Hungary; 2 Department of Family Medicine Semmelweis University Budapest Hungary; 3 The Medical Futurist Institute Budapest Hungary

**Keywords:** artificial intelligence, attitudes, health care professionals, digital health, fear, anxiety, reluctance, resistance, skepticism

## Abstract

**Background:**

The rapid progress in the development of artificial intelligence (AI) is having a substantial impact on health care (HC) delivery and the physician-patient interaction.

**Objective:**

This scoping review aims to offer a thorough analysis of the current status of integrating AI into medical practice as well as the apprehensions expressed by HC professionals (HCPs) over its application.

**Methods:**

This scoping review used the PRISMA-ScR (Preferred Reporting Items for Systematic Reviews and Meta-Analyses Extension for Scoping Reviews) guidelines to examine articles that investigated the apprehensions of HCPs about medical AI. Following the application of inclusion and exclusion criteria, 32 of an initial 217 studies (14.7%) were selected for the final analysis. We aimed to develop an attitude range that accurately captured the unfavorable emotions of HCPs toward medical AI. We achieved this by selecting attitudes and ranking them on a scale that represented the degree of aversion, ranging from mild skepticism to intense fear. The ultimate depiction of the scale was as follows: skepticism, reluctance, anxiety, resistance, and fear.

**Results:**

In total, 3 themes were identified through the process of thematic analysis. National surveys performed among HCPs aimed to comprehensively analyze their current emotions, worries, and attitudes regarding the integration of AI in the medical industry. Research on technostress primarily focused on the psychological dimensions of adopting AI, examining the emotional reactions, fears, and difficulties experienced by HCPs when they encountered AI-powered technology. The high-level perspective category included studies that took a broad and comprehensive approach to evaluating overarching themes, trends, and implications related to the integration of AI technology in HC. We discovered 15 sources of attitudes, which we classified into 2 distinct groups: intrinsic and extrinsic. The intrinsic group focused on HCPs’ inherent professional identity, encompassing their tasks and capacities. Conversely, the extrinsic group pertained to their patients and the influence of AI on patient care. Next, we examined the shared themes and made suggestions to potentially tackle the problems discovered. Ultimately, we analyzed the results in relation to the attitude scale, assessing the degree to which each attitude was portrayed.

**Conclusions:**

The solution to addressing resistance toward medical AI appears to be centered on comprehensive education, the implementation of suitable legislation, and the delineation of roles. Addressing these issues may foster acceptance and optimize AI integration, enhancing HC delivery while maintaining ethical standards. Due to the current prominence and extensive research on regulation, we suggest that further research could be dedicated to education.

## Introduction

### Background

Recently, health care (HC) has undergone a cultural shift known as digital health, which has changed the dynamic between physicians and patients. With the rise of patient empowerment, individuals have been seeking greater involvement in shared decision-making when it comes to problems that impact their health or disease management. Simultaneously, HC professionals (HCPs) have transitioned from the conventional patriarchal and authoritarian system to assume new roles as mentors, coaches, and guides. They now aid patients in navigating the complex HC system [1].

The fast changes in HC over the past decade, coupled with technological breakthroughs and the emergence of artificial intelligence (AI), have given HCPs new opportunities and posed unprecedented challenges. However, the successful integration of AI into HC requires addressing the existing challenges faced by HCPs, notably those related to their workload and well-being [2].

The health and mental well-being of HCPs already paint a distressing picture, even though their welfare increases the quality of care, productivity, and patient satisfaction [3]. Due to physician shortages and the existing burden on HCPs, the amount of effort, time, and skills needed to adopt these innovations is particularly important [4-6].

It seems AI has the potential to ease the everyday job of HCPs by offering support in diagnostics, decision-making, data analysis, and especially administrative tasks [7]. In addition, the implementation of AI offers other benefits, such as improved consultations, personalized health guidance, thorough medical record analysis, precision medicine, custom treatment plans, and innovative drug discovery [8]. Implementing AI into evidence-based medicine necessitates explicit standards and laws to ensure the safe use of these advanced technologies. Without such measures, the medical community may perceive AI as a potential threat rather than an opportunity [9]. Given that the nature of digital transformation is cultural and practicing medicine is highly dependent on the use of technologies, it is crucial for HCPs to carefully consider how they embrace the advancements of AI [10]. Despite numerous studies examining the attitudes and opinions of HCPs toward this breakthrough technology, the literature falls short of delving deeper into the subject [11,12].

The response of HCPs to the increasing use of AI plays a crucial role in shaping its development and its contribution to the field of HC [13]. Recent research shows a mix of interest and concern among HCPs regarding the use of AI.

Concerns include fear of job displacement, replacement, erosion of professional identity, and the loss of expertise [14,15]. HCPs worry about losing control to AI, leading to deskilling and overreliance on technology [16,17]. They also dread the potential negative impact on physician-patient relationships and uncertainty about the future of HC [16].

Ethical issues, such as biases in AI algorithms and automation bias, are significant worries [18]. Technostress and alert fatigue add to the anxiety. Technostress, in the realm of medical AI and its implications for HCPs, refers to the physiological strain experienced by HCPs due to the introduction, implementation, or ongoing use of advanced AI technologies in their practice [19].

Despite the aforementioned issues, the general attitude of HCPs toward medical AI remains positive overall, despite consistently emerging concerns. They demonstrate curiosity and openness to participate in training related to this field as they foresee the use of AI as the future trajectory [20-22].

### Objectives

In this scoping review, we aimed to dive deeper into the causes and explanations of HCPs’ concerns about medical AI. We provide an in-depth analysis of the negative attitudes experienced by HCPs regarding the adoption of AI, with the objective of understanding precisely what impedes their integration into professional practice.

## Methods

We conducted an initial literature search to assess what negative attitudes emerge toward medical AI. As there are psychological factors that underlie attitudes toward AI tools [8], we attempted to find studies focusing on precisely defined types of attitudes on an attitude scale. Subsequently, we selected the 5 attitudes that yielded the highest number of relevant results in the academic literature.

We conducted a thorough exploration of the literature using a systematic approach to identify the attitudes included in the scale. Specifically, we searched for all synonyms and related terms for *fear* using a thesaurus of synonyms and related words. From these, we examined which terms yielded a significant number of relevant results in academic literature. On the basis of this analysis, we selected 5 key terms—fear, resistance, skepticism, reluctance, and anxiety—to represent the range of negative attitudes toward medical AI. These terms not only captured the overarching sentiment of apprehension but also provided a nuanced framework to examine the underlying reasons behind HCPs’ concerns about integrating AI into clinical practice.

Therefore, we used an attitude scale to characterize the negative attitudes of HCPs, ranging from mild to intense aversion. The final scale was as follows: skepticism, reluctance, anxiety, resistance, and fear.

Skepticism represents a mild, questioning stance toward medical AI, often characterized by doubt and a demand for evidence without strong emotional biases. Reluctance builds upon skepticism, introducing a hesitant and unwilling attitude, yet still lacking significant emotional engagement. As the continuum develops to anxiety, we notice the onset of emotional responses, where concerns about potential risks and uncertainties begin to manifest more prominently. Resistance denotes a more active and deliberate opposition to medical AI, stemming from perceived threats to professional autonomy or patient safety. This stage reflects a substantial increase in both cognitive and emotional involvement. Ultimately, fear represents the most extreme kind of aversion, characterized by intense emotional responses and a deep feeling of danger, often resulting in strong opposition or avoidance activities.

The 5 attitudes form a spectrum of aversion to medical AI, where each attitude can influence or evolve into another under certain conditions. Skepticism, as a mild, questioning stance, often represents the starting point of negative attitudes. If skepticism is not addressed through evidence or reassurance, it may progress to reluctance, characterized by hesitation and unwillingness to engage with AI.

As uncertainty builds, anxiety may arise, driven by fears about risks and the unknown. This stage marks a significant shift as emotional responses become more prominent. Resistance typically emerges when anxiety intensifies into active opposition, often rooted in perceived threats to autonomy or professional roles. Finally, fear, the strongest negative attitude, represents a culmination of unresolved concerns, where emotional responses dominate and can hinder constructive engagement.

These attitudes are interconnected, with feedback loops that can exacerbate concerns. For instance, prolonged skepticism without resolution may deepen anxiety, and resistance may reinforce fear through confirmation biases. Understanding these interactions allows for tailored interventions, such as addressing skepticism with evidence early on or providing psychological support to reduce anxiety and fear.

This hierarchical structure corresponds to accepted psychological theories that propose a progressive increase in the strength of negative attitudes and feelings. The cognitive dissonance theory by Festiger [23] posited that individuals experience psychological discomfort when holding conflicting cognitions. This discomfort can escalate as the conflict intensifies. In our scale, skepticism represented a mild cognitive dissonance, where HCPs questioned the efficacy or safety of medical AI. As we moved toward reluctance and anxiety, the dissonance increased, reflecting greater internal conflict and emotional engagement.

The protection motivation theory by Rogers [24] explained how people respond to threats based on perceived severity and vulnerability, coupled with their coping efficacy. Skepticism and reluctance can be seen as early responses where perceived severity and vulnerability are low. As these perceptions heighten, they give rise to anxiety and resistance, culminating in fear when the perceived threat becomes overwhelming and coping mechanisms are deemed insufficient.

The objective of this approach was to offer insights into both the obstacles to adopting AI and the methods by which these obstacles might be addressed. It enabled the creation of strategies that were responsive to the concerns and demands of HCPs, promoting a more efficient and ethically mindful incorporation of AI into HC procedures. While the purpose of our work was not to create a validated methodology or standardized scale, we developed the attitude scale to provide a comprehensive framework for understanding HCPs’ negative attitudes toward medical AI.

Then, we conducted a scoping review using the PRISMA-ScR (Preferred Reporting Items for Systematic Reviews and Meta-Analyses Extension for Scoping Reviews) guidelines. The search query was ((artificial intelligence [Title] OR AI[Title])) AND (fear [Title/Abstract] OR resistance [Title/Abstract] OR skepticism [Title/Abstract] OR reluctance [Title/Abstract] OR anxiety.

The inclusion criteria for articles were as follows: (1) articles had to focus on the application of AI in medicine or HC and (2) they had to be original studies or reviews. Articles were eliminated based on the following criteria: (1) if the searched words were mentioned in a peripheral or unrelated context or were unrelated to medicine and HC; (2) if studies were not specifically focused on medical AI but rather on AI in general, even if they briefly addressed the *medical uses* of AI; (3) the studies excluded were not original research articles or reviews but rather editorials, commentaries, book reviews, or news pieces; (4) the studies were also excluded if they were from non–peer-reviewed journals, as these journals may not adhere to the high standards of more reputable journals; and (5) studies without abstracts in English were excluded. Textbox 1 shows the inclusion and exclusion criteria.

**Inclusion and exclusion criteria.**

**Inclusion criteria**

- Article type: original research articles or reviews
- Language: articles with abstracts in English
- Focus: studies specifically focused on medical artificial intelligence in health care
- Publication type: peer-reviewed journal articles

**Exclusion criteria**

- Article type: editorials, commentaries, book reviews, or news pieces
- Language: articles without abstracts in English
- Focus: studies in which artificial intelligence was mentioned only in unrelated or peripheral contexts
- Publication type: non–peer-reviewed articles

A total of 217 entries were discovered from PubMed, with 1 (0.1%) being excluded before screening due to duplication. We reviewed 216 records, and after careful evaluation, we determined that 166 (76.9%) of them did not meet our criteria and were therefore excluded. Subsequently, 50 (23.1%) records were requested for retrieval. A total of 18 (36%) of the 50 records were eliminated from the analysis. Of these 18 records, 12 (67%) were excluded because the article type did not meet the specified criteria, while the remaining 6 (33%) studies were excluded because their focus was not on the medical use of AI.

To ensure a systematic and unbiased selection, 2 independent reviewers conducted the screening and eligibility assessment. Any discrepancies were resolved through discussion, and in cases of disagreement, a third reviewer was consulted. This multireviewer approach minimized the risk of selection bias and ensured that only studies meeting the predefined criteria were included in the final analysis. After carefully considering the specific inclusion and exclusion criteria outlined subsequently, 32 studies were ultimately chosen for the review.

## Results

### Overview

We extracted the following data from each included study: title, authors, journal, year of publication, the methods and study design, the examined attitude type, the mentioned concern, and the URL. Data were entered into a Microsoft Excel spreadsheet for analysis. The spreadsheet is provided in Multimedia Appendix 1. Table 1 is the shortened version of this spreadsheet; for better readability, it shows the following data of all the selected studies: title, authors, journal, year of publication, and the examined attitude type.

**Figure 1:**
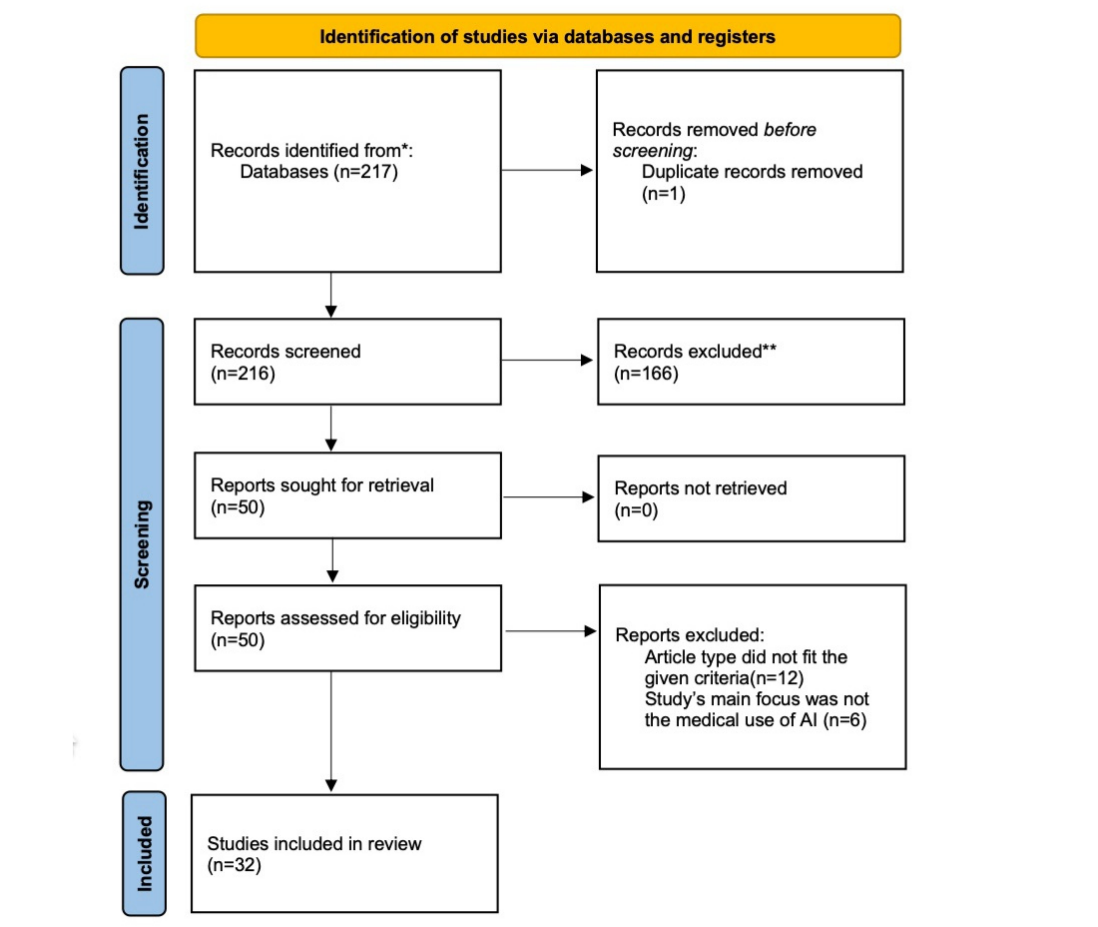
The PRISMA-ScR (Preferred Reporting Items for Systematic Reviews and Meta-Analyses Extension for Scoping Reviews) flow diagram illustrating the study selection process. The PRISMA-ScR flow diagram provides a visual summary of the study selection process. It details the number of studies identified (N=217), screened, assessed for eligibility, and included (n=32) in this scoping review. The diagram highlights the systematic approach to selecting relevant studies, ensuring transparency in the review methodology. AI: artificial intelligence. *Identified from PubMed.

The PRISMA-ScR flow diagram is shown in Figure 1. We identified 3 themes and assigned each selected study to one of those themes. The PRISMA-ScR checklist in the Multimedia Appendix 2.

The first theme was national surveys. National surveys (13/32, 40%) were comprehensive studies conducted on a nationwide level to evaluate the predominant emotions, concerns, and opinions of HCPs regarding the integration of AI in the medical domain. Studies in this category involved extensive surveys aimed at capturing a representative sample of HCPs from different specialties and regions within a specific nation or area, such as Jordan, Saudi Arabia, South Korea, Switzerland, or the United Kingdom. These investigations frequently used structured questionnaires and standardized methodologies to measure and analyze the attitudes, apprehensions, and perceptions of HCPs regarding the adoption of medical AI technologies.

The second theme was technostress. Studies (9/32, 9%) categorized under technostress primarily focused on the psychological dimensions of adopting AI, examining the emotional responses, anxieties, and challenges faced by HCPs when encountering AI-driven equipment. Studies in this field used qualitative methodologies, such as interviews or surveys, to capture intricate experiences and perceptions associated with technostress.

The third theme was high-level perspective. The high-level perspective category (10/32, 31%) included studies that adopted a broad and comprehensive approach to explore overall themes, trends, and consequences of integrating AI technologies in HC. Findings in this area encompassed systematic reviews, meta-analyses, and conceptual analyses that sought to consolidate and assess current research on medical AI while also incorporating fresh findings.

**Table 1:** Summary of studies included in this review. This table presents a condensed version of the detailed data provided in Multimedia Appendix 1. It lists the selected studies by title, first author, journal, year of publication, and the type of attitude examined. This summary facilitates a quick overview of the key studies and their primary focus within the scope of the review.

Title	Study	Journal	Study design
Applied artificial intelligence and trust—the case of autonomous vehicles and medical assistance devices	Hengstler et al [25], 2016	*Technological Forecasting and Social Change*	Case study
Influence of AI ethics awareness, attitude, anxiety, and self-efficacy on nursing students’ behavioral intentions	Kwak et al [26], 2022	*BMC Nursing*	Qualitative study
Diversity in people’s reluctance to use medical artificial intelligence: Identifying subgroups through latent profile analysis	Wang et al [27], 2022	*Frontiers in Artificial Intelligence*	Qualitative study
Is artificial intelligence the new friend for radiologists? A review article	Gampala et al [14], 2020	*Cureus*	Review
Artificial intelligence in modern medicine - the evolving necessity of the present and role in transforming the future of medical care	Bhattad and Jain [15], 2020	*Cureus*	Review
Artificial intelligence (AI) in medicine, current applications and future role with special emphasis on its potential and promise in pathology: present and future impact, obstacles including costs and acceptance among pathologists, practical and philosophical considerations. A comprehensive review	Ahmad et al [18], 2021	*Diagnostic Pathology*	Review
Artificial intelligence powers digital medicine	Fogel and Kvedar [28], 2018	*Nature Partner Journals Digital Medicine*	Review
Resistance to medical artificial intelligence	Longoni et al [29], 2019	*Journal of Consumer Research*	Review
Promise and provisos of artificial intelligence and machine learning in healthcare	Bhardwaj [30], 2022	*Journal of Healthcare Leadership*	Review
Exploring knowledge, attitudes, and practices towards artificial intelligence among health professions’ students in Jordan	Al-Qerem et al [31], 2023	*BMC Medical Informatics and Decision Making*	Qualitative study
Impact of artificial intelligence on US medical students’ choice of radiology	Reeder and Lee [32], 2022	*Clinical Imaging*	Qualitative study
Influence of artificial intelligence on Canadian medical students’ preference for radiology specialty: a national survey study	Gong et al [33], 2019	*Academic Radiology*	Qualitative study
Nursing students’ intent to use AI-based healthcare technology: path analysis using the unified theory of acceptance and use of technology	Kwak et al [34], 2022	*Nurse Education Today*	Qualitative study
Responsible use of artificial intelligence in dentistry: survey on dentists’ and final-year undergraduates’ perspectives	Roganović et al [35], 2023	*Healthcare (Basel)*	Qualitative study
Opinion research among Russian physicians on the application of technologies using artificial intelligence in the field of medicine and health care	Orlova et al [36], 2023	*BMC health services research*	Qualitative study
The integration of artificial intelligence in medical imaging practice: perspectives of African radiographers	Botwe et al [37], 2021	*Radiography*	Qualitative study
Perspectives of radiographers on the emergence of artificial intelligence in diagnostic imaging in Saudi Arabia	Aldhafeeri [38], 2022	*Insights Imaging*	Qualitative study
Perceptions of Canadian radiation oncologists, radiation physicists, radiation therapists and radiation trainees about the impact of artificial intelligence in radiation oncology - national survey	Wong et al [39], 2021	*Journal of Medical Imaging and Radiation Science*	Qualitative study
A qualitative study to explore opinions of Saudi Arabian radiologists concerning AI-based applications and their impact on the future of the radiology	Alsharif et al [40], 2022	*British Journal of Radiology Open*	Qualitative study
Healthcare professionals’ expectations of medical artificial intelligence and strategies for its clinical implementation: a qualitative study	Yoo et al [41], 2023	*Healthcare Informatics Research*	Qualitative study
The impact of artificial intelligence on clinical education: perceptions of postgraduate trainee doctors in London (UK) and recommendations for trainers	Banerjee et al [16], 2021	*BMC Medical Education*	Qualitative study
A survey on the future of radiology among radiologists, medical students and surgeons: students and surgeons tend to be more skeptical about artificial intelligence and radiologists may fear that other disciplines take over	van Hoek et al [42], 2019	*European Journal of Radiology*	Qualitative study
Acceptance of clinical artificial intelligence among physicians and medical students: a systematic review with cross-sectional survey	Chen et al [43], 2022	*Frontiers in Medicine*	Review
Artificial intelligence and employee’s health - new challenges	Walusiak-Skorupa et al [44], 2023	*Medycyna Pracy Workers’ Health and Safety*	Review
Artificial intelligence in anesthetic care: a survey of physician anesthesiologists	Alamo et al [45], 2024	*Anesthesia and Analgesia.*	Qualitative study
Increasing acceptance of medical AI: The role of medical staff participation in AI development	Huo et al [46], 2023	*International Journal of Medical Informatics*	Qualitative study
Impact of the rise of artificial intelligence in radiology: what do students think?	Barreiro-Ares et al [47], 2023	*International Journal of Environmental Research and Public Health.*	Qualitative study
An international survey on AI in radiology in 1,041 radiologists and radiology residents part 1: fear of replacement, knowledge, and attitude	Sangers et al [48], 2023	*Archives of Dermatological Research*	Qualitative study
Towards successful implementation of artificial intelligence in skin cancer care: a qualitative study exploring the views of dermatologists and general practitioners	Huisman et al [49], 2021	*European Radiology*	Qualitative study
Identity threats as a reason for resistance to artificial intelligence: survey study with medical students and professionals	Jussupow et al [17], 2022	*JMIR Formative Research*	Qualitative study
Acceptance and resistance of new digital technologies in medicine: qualitative study	Safi et al [50], 2018	*JMIR Research Protocols*	Qualitative study
Knowledge and attitudes towards artificial intelligence in imaging: a look at the quantitative survey literature	Bhandari et al [51], 2021	*Clinical Imaging*	Review

### Theme 1: National Studies

There were 13 studies in this category, all of which were quantitative studies. These publications conducted a focused analysis of the attitudes of physicians or other HCPs working in a particular country or area, and the results were specific to that geographic area.

One of the Saudi Arabian studies identified multiple factors that prevented radiologists from using AI. These barriers included budgetary constraints, absence of regulations, insufficient support, inadequate training, and lack of AI-based apps and expertise. The lack of expertise, regulatory laws, and support systems were cited as major barriers to the effective adoption of AI in another article. Radiographers struggled to obtain AI-related training [38,40].

Researchers in Jordan identified similar issues. In their studies, the barriers included not knowing enough about AI, having insufficient access to AI or to the right technical equipment, concerns about ethics and privacy, not having enough time due to educational commitments, AI being perceived as too complicated, not learning about AI as part of the educational curricula, and not having places or opportunities to learn and practice AI skills [31].

Researchers in Africa discovered comparable anxieties. Radiographers indicated that the introduction of AI and its use could potentially have a negative impact on their core skills. Issues of ethics and potential medico-legal concerns in relation to image manipulation and cybersecurity were also highlighted. Reported issues included insufficient worker training and technological competence, inadequate data-right frameworks, public policy limitations, high costs associated with AI equipment installation and management, concerns about employment displacement, and difficulty with internet connectivity [37].

Gong et al [33] conducted a study on the impact of AI on the level of interest among Canadian medical students in the field of radiography. The apprehension regarding the potential displacement of radiologists by AI, rather than their complete replacement, dissuaded numerous students from contemplating a career in radiology. Another Canadian study on oncology professionals showed similar findings—moderate AI knowledge and concerns about job loss and practice changes due to AI [39].

Kwak et al [26] conducted a study in South Korea to investigate the views toward behavioral intents of nursing students. Most nurses and students (>70%) lacked comprehension of AI in clinical practice. Their concerns pertained to discrimination and ethical issues caused by malfunctions and incomplete technology of AI medical devices as well as the distortion and bias of information due to lack of accumulated data or learning errors in AI and invasion of privacy. AI ethics awareness did not significantly influence behavioral intention.

The same researchers surveyed 210 nursing school students in South Korea to predict their intent to use AI-based HC technologies. They found that performance expectancy and self-efficacy had a negative effect on the path to negative attitude, whereas anxiety had a positive effect. In addition, they pointed out that >70% of the students did not understand AI, and there were growing concerns around discrimination, privacy, ethical issues caused by malfunctions, and distortion and bias of information due to a lack of accumulated data or learning errors in AI [34].

Another South Korean study examined HCPs at the emergency and intensive care unit of a tertiary teaching hospital in Seoul. Participants mentioned concerns regarding distortions in the workflow, deskilling, alert fatigue, and unsophisticated algorithms. If medical AI decisions contradicted their judgment, most participants would consult other medical staff and thereafter reconsider their initial judgment [17,41].

Banerjee et al [16] studied postgraduate trainee physicians’ perceptions in London, United Kingdom. A thematic analysis of free-text responses revealed negative subthemes. These included less development of clinical judgment, limited opportunity for practical skills, workflow intrusion, an increased administrative workload, and diminished development of clinical accountability and probity. Trainees believed that clinical AI might reduce their practical skills, clinical judgment, and decision-making. Another concern was the increased administrative workload potentially causing information overload.

According to the survey by Orlova et al [36], 35.6% of the respondents in Russia indicated that they were familiar with AI. Among the challenges, respondents cited a lack of flexibility and limited application on controversial issues. In addition, they expressed concerns about AI decision-making difficulty with insufficient information, and they feared the involvement of inexperienced specialists in AI development. Moreover, 89% of the respondents believed HCPs should be involved in AI development for medicine and HC. Ethical and legal concerns and lack of knowledge were commonly cited obstacles to AI implementation.

Roganović et al [35] performed a survey among proficient dentists and senior undergraduate students from the School of Dental Medicine at the University of Belgrade, Serbia. A total of 193 respondents, particularly students, showed a deficiency in understanding AI and expressed skepticism toward it. The primary factors contributing to this situation were the lack of knowledge about AI technology associated with a fear of being replaced by AI as well as the absence of regulatory policy. Female dentists exhibited a higher level of awareness and concern regarding ethical considerations related to the application of AI in dental practice compared to their male counterparts.

van Hoek et al [42] focused their attention on radiologists, students, and surgeons in Switzerland. The survey had 170 participants. Surgeons had a lower level of supportiveness compared to radiologists (*P*=.001). Students saw a potential threat of AI as more likely than radiologists did (*P*=.04). Medical students and surgeons tended to be more skeptical about AI; students saw AI as a potential threat to diagnostic radiologists, while radiologists themselves were rather afraid of turf losses.

### Theme 2: Technostress

The term *technostress* was specifically chosen for this theme because it accurately encapsulated the unique stressors and psychological burdens associated with the integration of advanced technologies into professional workflows. There were 9 studies, consisting of 7 (78%) qualitative research investigations and 2 (22%) literature reviews. On the basis of qualitative research, 4 (44%) studies investigated the perspectives of HCPs toward medical AI, irrespective of their specialization; 1 (11%) study specifically focused on radiologists, another (11%) on anesthesiologists, and 1 (11%) explored the opinions of dermatologists and general practitioners (GPs).

Dermatologists and GPs first identified the main barriers to the use of AI as doubts about the accuracy of AI, lack of integration of clinical findings in the assessment of an algorithm, and lack of algorithm transparency. The second main barrier was the risk of health inequalities, such as bias toward light skin types in algorithm training data and accuracy deviations between hospitals when (not) using AI. The third barrier mentioned by dermatologists was the fear of being replaced by AI. GPs mentioned the extra time it will take to use AI [48]. The fear of replacement was also mentioned in the international survey on AI in radiology; 38% of the participants emphasized it. Male participants with little AI-specific knowledge reported fear at a considerably higher frequency. Intermediate and advanced knowledge levels might enhance the adoption of AI in clinical practice, while rudimentary knowledge levels appeared to be inhibitive. Career doubt was significantly associated with fear of replacement in 95% of the people (*P*<.001) [49].

The findings aligned with research that investigated the perspectives of anesthesiologists. In addition, 45% of the participants voiced apprehension regarding the potential impact of incorporating medical AI on the need for anesthesiologists and their income. They believed that within a decade, AI would surpass them in predicting adverse perioperative events (83%), formulating pain management plans (67%), and conducting airway examinations (45%). The main barriers identified were the lack of algorithmic transparency (60%), an environment conducive to malpractice claims (47%), and the potential for medical errors (47%) [45].

There were 4 studies in the technostress category, which investigated the emotions of HCPs regardless of their medical specialization. They all obtained comparable findings. The research by Safi et al [50] validated that the use of novel technology in the HC sector was contingent upon individual characteristics. HCPs feared that technology was a means of management control and thereby hindered their autonomy in making diagnoses and impacted their interactions with patients. Jussupow et al [17] examined if certain apprehensions related to the new technology could pose a significant enough risk to result in its rejection and opposition. The study investigated 2 aspects of medical professional identity threat: challenges to the physician’s expertise and challenges to the physician’s role as an independent HCP. Both threats contributed to perceived self-threat and resistance to AI. Medical students experienced stronger identity threats and resistance to AI than HCPs.

Huo et al [46] investigated whether the participation of HCPs in the development of AI decreased opposition to AI. They demonstrated the psychological impact of staff participation on the acceptability of AI. They distinguished between AI for individual diagnosis and AI for assistive diagnosis, suggesting that AI anxiety mediated staff participation’s impact on AI acceptance. They found a positive effect of staff involvement on AI for individual diagnosis and AI for assistive diagnosis acceptance. Cognitive and affective attitudes both influenced AI acceptance. Speciesism moderated the effect of staff participation on AI anxiety; staff with higher speciesism levels tended to reduce HCP’s anxiety when they were involved in AI development.

Chen et al [43] discovered that both HCPs and medical students expressed several concerns regarding medical AI. These concerns included a lack of trust in clinical AI and a preference for results to be verified by human clinicians. There were also worries about the unpredictability of AI results and the potential for errors in clinical AI. In addition, there were concerns about operator dependence and the increased procedural time caused by the use of medical AI. Other concerns included the poor performance of AI in unexpected situations, its lack of empathy or communication, the absence of ethically defensible laws and policies, the ambiguity surrounding medico-legal responsibility for errors made by AI, data security and the risk of privacy breaches, the opaque nature of AI algorithms, the limited availability of high-quality datasets for training and validation, and a shortage of interdisciplinary talent. The two systematic literature reviews mainly emphasized the significance of education.

The results of the study by Bhandari et al [51] showed that students were more anxious about future job prospects than HCPs. Moreover, 48.2% of the students were less likely to choose radiology as a career because of AI. Walusiak-Skorupa et al [44] examined the influence of AI on the overall well-being of workers. The negative impacts might manifest in both physical and psychological aspects. Physically, it could result in a lack of proper machine control and an increased risk of accidents. Psychologically, it could lead to technostress, fear, employment exclusion due to automation, changes in the labor market, and the widening of social disparities. Within the HC domain, our primary focus lay on addressing the psychological impacts.

### Theme 3: High-Level Perspective

This perspective examined broad patterns, ethical concerns, worldwide trends, and possible societal effects, offering a comprehensive view of the subject matter. The research mentioned subsequently contributed valuable insights into the broader discourse surrounding medical AI, informing policy discussions and future research directions.

Consumers who had a better perception of their own uniqueness tended to exhibit greater resistance to medical AI. HCPs expressed concerns about the ability of medical AI to effectively handle the individual characteristics of patients, which might result in a reluctance to adopt medical AI in practical settings. Barreiro-Ares et al [47] examined the impact of the rise of AI in radiology. From the 281 respondents, 95.7% agreed with the need to implement well-established ethical principles in AI. The biggest concern was that they could not interpret the patient in a global clinical context (78.65%); this was in line with the results of the previous study. Other concerns were the high-cost implementation (41.28%), the possible vulnerability of the right to privacy of patients (38.08%), and necessary training in the management of AI for HCPs (38.08%) [36,41]. Bhardwaj [30] also found that the caveats and challenges with the use of AI were data acquisition and validation, a paradigm shift in patient care, cost-benefit and value proposition, data ownership and integrity, and issues surrounding consumer privacy [52].

Another study also analyzed people’s hesitancy to use medical AI and found a gap in existing research. Researchers shifted from a variable-centered approach to a person-centered approach. They discovered that while some individuals consistently held certain knowledge, attitudes, and behaviors toward medical AI, others showed more variability. According to them, there might be a disconnect between one’s knowledge and a negative attitude toward medical AI, and decision makers should be cautious when advising individuals, even those with high knowledge of medical AI [27]. Certain professions, such as radiology, have embraced AI quite quickly, while others, particularly pathology, are just starting to incorporate AI into their practices. Within the community of radiologists, there were certain disadvantages and prejudices about medical AI.

The disadvantage was that AI necessitated a substantial amount of data to create high-quality training sets for algorithms to acquire knowledge. This process could be costly and time-consuming for radiologists. The extensive adoption of the new technology could amplify systemic risks of harm, elevate the probability of errors with substantial consequences, and intensify complex societal and ethical concerns. Pathologists had concerns about the possibility of being substituted by computer algorithms. They emphasized that regulatory approval was essential [18].

A crucial subject of the discussion revolved around enhancing trust in AI. Researchers examined applied AI and trust in the case of autonomous vehicles and medical assistance devices. Our focus was on the latter topic. They suggested that firms must begin to build trust during the development process of a technology. In the ever-digitalizing world of medicine and its diverse branches, HCPs need to support AI rather than fear replacing skilled medical professionals [15,25].

The integration of AI is reshaping the roles and competencies of HCPs, fostering new forms of professional identity that blend human expertise with technological augmentation, which manifests intrinsically. Extrinsically, it enhances diagnostic and therapeutic accuracy while simultaneously introducing ethical and empathetic challenges that redefine the dynamics of the patient-clinician relationship. In summary, across the 3 themes, we identified 15 specific sources of attitudes mentioned in the studied literature.

For better comprehensibility, we divided these sources into 2 groups, based on where the attitude was coming from—intrinsic and extrinsic groups. The first group was about HCPs’ own professional identity concerning their roles and capabilities, while the other was related to their patients and the impact of AI on patient care. Extrinsic sources contained external factors, such as organizational policies, ethical issues, patient connections, and technological limitations, while intrinsic sources contained internal factors, such as personal beliefs, skills, personal identity, and expert status.

**Figure 2:**
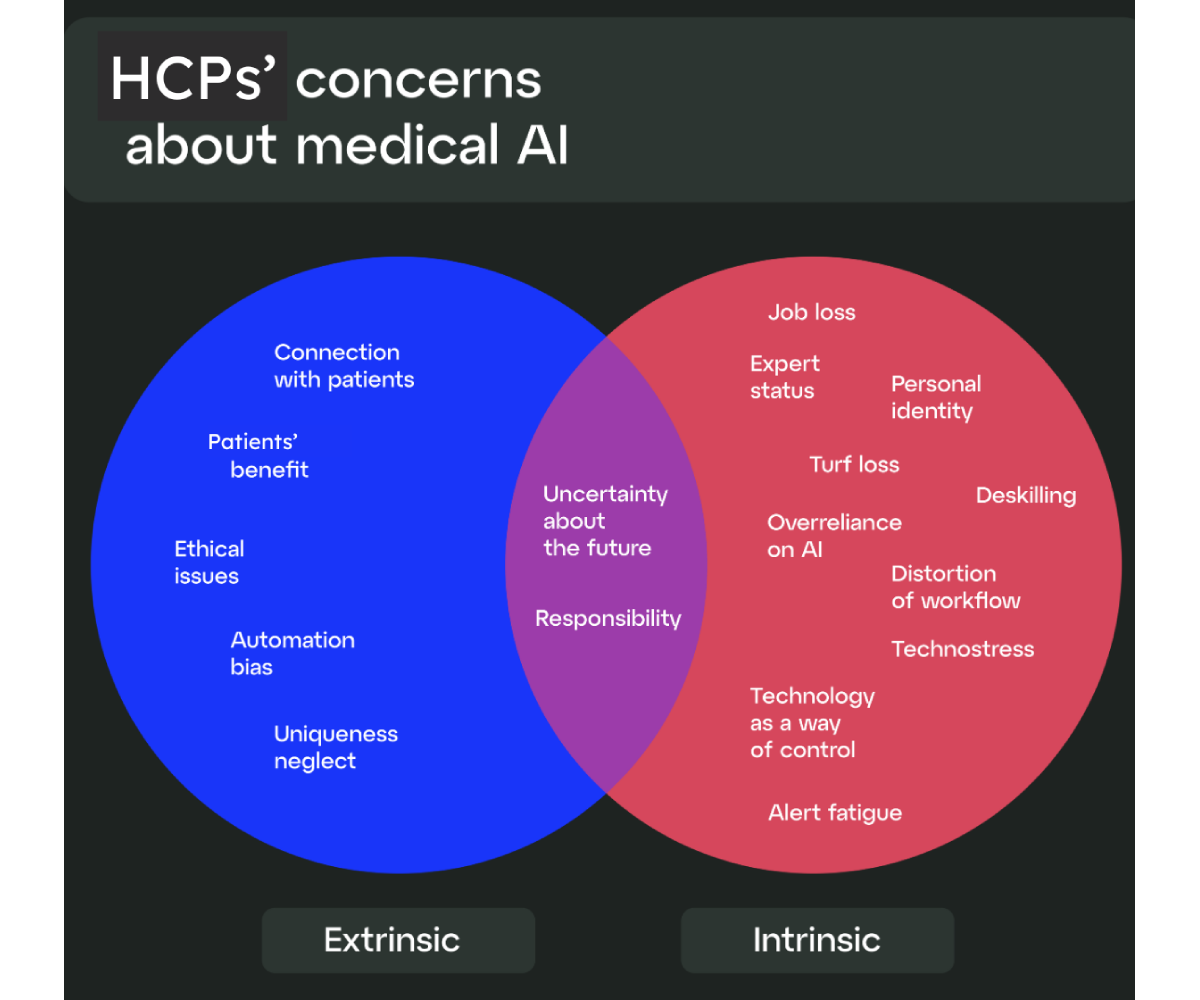
Classification of sources of attitudes among health care professionals (HCPs) as identified in the review. This figure categorizes the 15 identified sources of attitudes of HCPs related to the use of medical artificial intelligence (AI). The sources of attitudes are divided into 2 main categories: extrinsic (external factors such as organizational policies, ethical issues, patient connections, and technological limitations) and intrinsic (internal factors such as personal beliefs, skills, personal identity, and expert status). Two sources, uncertainty about the future and responsibility, are shown to overlap, affecting both HCPs and their patients. The classification aids in understanding the multifaceted nature of these concerns.

Two of the sources, uncertainty about the future and responsibility, were shown to overlap, affecting both HCPs and their patients. This classification, as shown in Figure 2, aided in understanding the multifaceted nature of these sources of attitudes.

## Discussion

### Principal Findings

This scoping review examined the negative attitudes of HCPs toward medical AI to understand the barriers to its adoption. We identified 5 key attitudes: skepticism, reluctance, anxiety, resistance, and fear. Thematic analysis revealed 3 main themes: national surveys, technostress, and high-level perspectives. Analyzing the 32 studies in our scoping review allowed us to examine the attitude scale mentioned earlier and identify the potential sources of those attitudes. While HCPs recognize AI’s potential, they also express apprehension driven by fear. This study explores the sources of these negative attitudes. Addressing these through education, HCP involvement in AI development, and clear regulations may facilitate successful integration.

We interpret our findings within an international context to provide a broader understanding of their significance. In the intrinsic group, technostress emerges as a key concept and source of negative attitudes. Technostress manifests as anxiety, tension, or distress from feeling overcome by using new technology. This feeling appears when HCPs struggle to adjust to and master the new technology positively and efficiently. This can lead to hesitation toward the integration of medical AI in their daily practice.

The term *technostress* was developed in 1984, as psychologist Craig Brod introduced it in his book *Technostress: The Human Cost of the Computer Revolution*. Brod describes technostress as a condition resulting from our inability to adapt to new computer technology. It manifests either in an overidentification with computers or computer anxiety. Technostress influences individuals’ orientation to time, communication modes, and interpersonal relationships [53]. Currently, there are 5 distinct subcategories of technostress.

Techno-burden refers to the feeling of being overwhelmed by excessive data and constant notifications. Techno-intrusion occurs when the lines between personal and professional life blur due to expectations of constant connectivity. Techno-complication involves confusion about effectively using technology. Techno-ambiguity arises from the uncertainty caused by rapid digital changes. Finally, techno-insecurities involve concerns about job displacement or changes in roles due to the introduction of new technology [43]. We encompass all subcategories within the umbrella term of technostress.

In contrast to intrinsic sources of attitudes, extrinsic sources refer to HCPs’ worries about how the implementation of medical AI will impact the health and well-being of their patients as well as the dynamics of their relationships with them. This aspect also encompasses ethical and societal considerations.

Ethical problems related to medical AI have been a concern of researchers for a long time. The first systematic review of studies that compared forecasts made by humans (clinical judgments) to forecasts made by statistical models was published by Meehl [54]. Their review demonstrated that statistical models outperformed people in predicting a range of outcomes. During a comparison conducted by UK researchers, the accuracy of triage diagnoses provided by HCPs was found to be 77.5%, whereas AI achieved a higher accuracy rate of 90.2%.

HCPs generally prefer to rely on their own intuition rather than on statistical models and are evaluated as less professional and less competent if they do rely on computerized decision aids. Moreover, people are more likely to follow the recommendation of a physician than the recommendation of a computer [29].

This can be explained by a phenomenon called *uniqueness neglect*, which refers to the apprehension that AI providers are less capable than human providers in considering the distinctive traits and situations of consumers. In our context, consumers can refer to both HCPs who are concerned about their patients as well as the patients themselves who have apprehensions about the ability of medical AI to accurately identify and effectively address their individual medical conditions. While customers perceive themselves as distinct and separate from others, they perceive machines as being capable of functioning only in a standardized and repetitive manner that addresses every task or position in an identical manner. Situations that threaten self-uniqueness result in feelings of anxiety.

In the HC context, these beliefs manifest in consumers viewing their health-related characteristics as unique and distinct from those of an average person. Uniqueness neglect drives consumer resistance to medical AI from both HCPs’ and patients’ sides [29]. Extrinsic sources of attitudes predominantly appeared within the high-level perspective theme among the 3 themes. However, some research also mentions extrinsic sources in the other 2 themes [34,43,44,51].

As we briefly mentioned previously, two sources of attitudes, responsibility and uncertainties about the future, belong to both categories, as they affect both the HCPs themselves and their patients.

Responsibility in medical AI is complex and involves multiple stakeholders. When issues arise, accountability could fall on the technology developer, the employing institution, the physician using the AI, or the patient who has given consent to its use. The assignment of responsibility depends on the specific context. The uncertainty regarding the future also equally affects HCPs in their practice, as they may fear whether AI will eventually replace them or if they can keep pace with rapidly evolving technology. This uncertainty also extends to patients. HCPs may be concerned about what the future holds for their patients and whether the use of new technology will undoubtedly benefit them. Sources of attitudes found at the intersection are exemplified by all 3 themes in nearly equal proportions.

The examined scale of the 5 attitudes encompassed a wide spectrum of concerns. The frequency of these attitudes reported in the literature is as follows: skepticism in 19% (6/32) of the studies, reluctance in 2% (2/32) of the studies, anxiety in 25% (8/32) of the studies, resistance in 9% (3/32) of the studies, and fear in 41% (13/32) of the studies.

The attitude scale, organized from the weakest to the strongest negative attitudes, includes skepticism, reluctance, anxiety, resistance, and fear. Notably, the strongest negative attitude, fear, was most frequently reported across the studies. We also examined which specific sources are associated with each type of attitude. It appears that fear, anxiety, skepticism, and resistance include both extrinsic and intrinsic types of concerns, whereas reluctance encompasses exclusively intrinsic concerns. As depicted in Table 2, intrinsic sources are much more comprehensive, appearing in almost every attitude, while extrinsic sources are only present in 1 or 2. Intrinsic sources, such as job loss and personal identity, significantly influence all measured attitudes, reflecting their broad impact. Conversely, extrinsic sources of attitudes, such as ethical issues and patient benefits, show a narrower influence. The figure’s visualization facilitates an understanding of how individual sources of attitudes align with specific professional attitudes.

**Table 2:** Association between health care professionals’ sources of attitudes and the attitude scale. This figure illustrates the relationship between specific sources of attitudes and the attitudes of health care professionals, as measured by the attitude scale. The figure’s visualization facilitates an understanding of how individual sources of attitudes align with specific professional attitudes.

Sources		Skepticism	Reluctance	Anxiety	Resistance	Fear
**Intrinsic**						
	Job loss	✓	✓	✓		✓
	Personal identity	✓	✓	✓	✓	✓
	Expert status	✓	✓	✓	✓	✓
	Turf loss and authority	✓	✓	✓	✓	✓
	Deskilling	✓			✓	
	Overreliance on AI^a^				✓	
	Distortion of workflow	✓		✓		
	Technostress	✓	✓	✓	✓	✓
	AI as a way of control				✓	
	Alert fatigue	✓				
**Extrinsic**						
	Patient benefit			✓		✓
	Ethical issues			✓		✓
	Automation bias					✓
	Uniqueness neglect				✓	
	Connection with patients	✓			✓	✓
**Common**						
	Uncertainty			✓	✓	
	Responsibility	✓	✓	✓		✓

^a^AI: artificial intelligence.

One of the aims of our research is to offer resolutions for the challenges we have examined. Our findings suggest that the following factors may facilitate the effective medical application of AI: accessible and high-quality education, the involvement of HCPs in the development process, the understanding of HCPs’ sources of attitudes, and transparent regulation of medical AI.

Education has the potential to ease the worries that HCPs have about themselves. The current findings indicate that the more they know about medical AI, its operations, potential, and limitations, the more confident they become and the more willing they are to incorporate it into their daily practice. Regarding the ethical and social concerns related to patients, we see the possibility of mitigation through the establishment of appropriate regulations. The strategies mentioned subsequently might help mitigate the concerns of HCPs in the future.

In order to effectively integrate AI-based technology into clinical practice, HCPs and students have recommended improving the system’s accuracy, progressively incorporating AI systems into routine procedures, and developing and sustaining confidence among HCPs through the accumulation of data. They emphasized that machine learning should be performed using sufficient input variables to develop algorithms and that training datasets should contain absolutely no errors. The importance of alert fatigue management and integration into workflow was emphasized. AI-based methods can be used to optimize medication alerts in a hospital setting [55].

Furthermore, legal issues about medical decision-making based on algorithms and manageable costs for system integration and the use of algorithms were mentioned [35,36]. It seems that best practices should involve collaboration among developers, HC institutions, and physicians to ensure the safe and responsible use of AI. This collaborative approach would help mitigate risks and enhance the reliability and effectiveness of medical AI systems.

On the basis of our findings, the acceptance of digital solutions and innovative medical technology by HCPs relies on understanding their anxieties and feelings of insecurity. Education is the key element in the acceptance of medical AI and the reduction of concerns associated with it for both medical students and HCPs.

The more education and experience a physician has with medical AI, the more adept they are at overcoming obstacles and the more confident they become. All studies agree that attitudes and knowledge about AI in the medical field remain a topic that needs further research and education regarding its use in the clinical setting.

According to Chen et al [43], medical AI should be incorporated into medical training curricula and postacademic training. HCPs need to learn how to operate AI tools, judge the reliability of AI results outputs, and redesign current workflows. Furthermore, HCPs should not only become primary AI users but also should be involved in the construction of AI technologies.

Medical students appear to be interested in AI, but they have not received education about AI and do not feel they understand its basic computational principles or limitations. AI appears to have a current deficit in the medical curriculum. These results are consistent with previous surveys conducted internationally [56].

However, the exact method of this education is crucial. There are many training programs offered by a wide range of institutions, but they are often occasional, short, and not integrated into the learning path of HCPs. The fact that AI training is only recently emerging creates a significant gap between what these programs offer and what HCPs need to learn. What is clear is the need for adequate training that includes the use, benefits, challenges, and issues related to the implementation of AI in clinical departments to ensure that it increases the confidence of HCPs.

The findings also suggest the need for education in data literacy, technical literacy, system thinking, AI algorithms, and AI’s ethical meaning to improve HCPs’ competency. Without aiming for completeness, we mention some educational programs that are already available, as shown in Table 3.

**Table 3:** Already available educational programs about the use of medical artificial intelligence (AI).

Program title; source	References
AI for Healthcare: Equipping the Workforce for Digital Transformation; FutureLearn	[57]
Artificial Intelligence in Health Care; MIT Management Executive Education	[58]
Digital Health; Harvard Online	[59]
AI and Digital Transformation in Healthcare; University of Cambridge Institute of Continuing Education	[60]
Foundations of AI in Healthcare; University of Melbourne	[61]

Barreiro-Ares et al [47] suggested that students should participate in hands-on activities that involve real-world applications of AI. They should also learn to use AI effectively and critically in their work. Similarly, younger individuals with high potential should be provided with programs that enable them to strategically plan and advance their professional careers for the future.

Even though most of the research discussed emphasizes the beneficial side of education in relation to medical AI, according to the study by Banerjee et al [16], medical educators should note that there are some areas in which clinical AI could potentially hinder training. Involving HCPs in the development of algorithms will help trainees continue to develop their skills because then AI will depend on their training to mimic behavior. This will also increase trust in AI technologies and improve explainability to patients.

According to Bhardwaj [30], concerns over consumer privacy and ownership of vast amounts of HC data in an AI-driven landscape seem to be crucial topics. Both intentional and unintentional breaches might result in financial damage. Policy makers and legislative bodies need to establish regulations defining the role of third-party payers in financing machine learning–assisted HC.

Public education and training through various media channels are essential to address misperceptions about machine learning–assisted algorithms and shape public opinion. According to the European and North American multisociety statement, AI should prioritize human rights, privacy, and dignity to foster trust between patients, HCPs, and AI systems. However, a broad definition of transparency must balance revealing critical information with protecting patient privacy [30].

Given the current severe shortage of HCPs and the excessive workload they face, there is a critical need for the support AI can provide HCPs. Using medical AI, HCPs can outsource repetitive, data-based tasks; promote their well-being; and ensure they can provide optimal care to patients [14]. When HCPs effectively and confidently use technological support, they can simplify repetitious, frequently monotonous, and demanding administrative work, allowing them to prioritize their main purpose in choosing this profession, that is, providing patient care and passionate healing [62,63].

A scoping review is susceptible to several limitations. A significant constraint is the presence of selection bias. If the studies selected are not representative of the full corpus of pertinent research, it can result in potentially biased results. In addition, our work may be subject to data-driven bias, as we specifically chose the attitudes that comprised the scale, which may not include all the relevant attitudes experienced by HCPs. The search was constrained to specific keywords (fear, resistance, skepticism, reluctance, and anxiety), perhaps excluding pertinent publications that used other vocabulary to depict negative sentiments toward AI.

Only articles that have abstracts in English were considered, potentially excluding relevant studies published in other languages. This review exclusively relied on PubMed as a source, perhaps overlooking pertinent studies cataloged in other databases.

Another constraint is the possibility of publication bias, wherein research with favorable results is more inclined to be published, while studies with unfavorable or inconclusive results may be inadequately represented, thus potentially restricting the available selection of articles. By excluding editorials, commentaries, and other nonprimary research articles, significant insights and expert viewpoints may have been overlooked.

The subjective interpretation of qualitative data can lead to researcher bias, as our perceptions may impact the categorization and analysis of negative sentiments. Differences in the definition and measurement of unfavorable attitudes in various research may impact the ability to compare the results.

Finally, the rapid evolution of AI in medicine means that our findings may quickly become outdated as new technologies and insights emerge, limiting the long-term applicability of our conclusions.

Considering all these factors, we still believe that our findings provide a valuable contribution to understanding and addressing the negative attitudes of HCPs toward medical AI. While previous studies have extensively examined general attitudes toward medical AI, our findings highlight a critical gap, as the literature has neglected the specific negative attitudes of HCPs.

Our findings extend beyond the immediate challenges of AI adoption, highlighting a broader issue in the digital and cultural transformation of HC—the need to reconcile technological advancements with the professional identity and ethical responsibilities of HCPs. The negative attitudes toward AI are not solely reactions to the technology itself but reflect deeper doubts regarding autonomy, ethical questions, clinical decision-making, and the changing role of HCPs in patient care.

Successful AI integration requires a shift in perception, positioning AI as a tool that augments, rather than replaces, human expertise. This necessitates structured AI education within medical curricula, continuous professional development, interdisciplinary collaboration in AI development, and the establishment of transparent ethical and regulatory frameworks. Addressing these negative attitudes is critical for fostering AI acceptance and ensuring that technological progress in HC remains aligned with the best interests of both patients and the HCPs. Beyond education and regulatory efforts, AI adoption strategies must also acknowledge and actively mitigate these deeply rooted negative attitudes, ensuring that AI integration does not erode HCPs’ sense of expertise or professional identity.

### Conclusions

In our scoping review, the studies selected for the final analysis were classified into 3 themes: national, technostress, and high-level perspective. We developed an attitude scale to examine concerns related to medical AI as reported in the literature. The attitude scale appears to be well-covered, and numerous sources of attitudes have also emerged. These sources were categorized into 2 groups based on their origin: extrinsic and intrinsic.

We analyzed the association between these sources of attitudes and the 3 identified themes as well as the 5 attitudes from the scale. It appears that fear, anxiety, skepticism, and resistance include both extrinsic and intrinsic sources, whereas reluctance encompasses exclusively intrinsic ones. Intrinsic sources of attitudes are much more comprehensive, appearing in almost every attitude, while extrinsic sources of attitudes are only present in 1 or 2 attitudes.

In summarizing the findings, we conclude that the key to overcoming resistance toward medical AI seems to lie in effective education and the establishment of appropriate regulations, along with the clarification of responsibilities. Given that regulation is currently a trending topic and the subject of numerous studies, we chose to focus more on education [52,64-66].

There are currently diverse training programs available on this topic, even though their quality varies. We have mentioned some education programs in the Discussion section. It is crucial for physicians to be able to differentiate which sources provide trustworthy knowledge about the use of medical AI. In addition, it is also important to draw decision makers’ attention to the existing educational programs and to take advantage of the opportunities they offer.

Our findings aim to offer valuable insights for enhancing communication between HCPs and patients while providing HC institutions with novel prospects for the advancement of AI-based technology. We hope that the findings can make a substantial contribution to the continued development of AI-driven technologies in HC, empowering HCPs to deliver more individualized and efficient treatments, resulting in increased happiness and a heightened sense of proficiency in their profession.

## Appendix

Multimedia Appendix 1 Detailed information on the studies analyzed in this review: title, first author, journal, year of publication, methods and study design, type of attitude examined, specific concerns mentioned, and direct URLs to the studies.

Multimedia Appendix 2 PRISMA-ScR checklist.

## Abbreviations

AI: artificial intelligence
GP: general practitioner
HC: health care
HCP: health care professional
PRISMA-ScR: Preferred Reporting Items for Systematic Reviews and Meta-Analyses Extension for Scoping Reviews

## Data Availability

All data generated or analyzed during this study are included in this published article and its supplementary information files.
